# Speckle tracking derived strain in infants with severe perinatal asphyxia: a comparative case control study

**DOI:** 10.1186/1476-7120-11-34

**Published:** 2013-09-03

**Authors:** Arvind Sehgal, Flora Wong, Samuel Menahem

**Affiliations:** 1 Monash Newborn, Monash Medical Centre, Melbourne, VIC 3168, Australia; 2 Department of Pediatrics, Monash University, Melbourne, Australia; 3 MonashHeart, Southern Health, Melbourne, Australia

**Keywords:** Asphyxia, Neonates, Speckle tracking, Strain

## Abstract

**Background:**

Speckle tracking echocardiography is increasingly being used to assess cardiac function in neonates. The objective was to compare speckle tracking strain indices between asphyxiated infants and healthy controls and to ascertain correlations between strain and 2D Doppler derived indices and cardiac troponin (biochemical marker of myocardial injury).

**Methods:**

Clinical and echocardiographic data from severely asphyxiated infants undergoing therapeutic hypothermia was evaluated retrospectively. This was compared with prospective data from healthy infants. Correlations between variables were assessed using Pearson’s coefficient of correlation.

**Results:**

Twenty four infants with severe perinatal asphyxia were admitted during the study period of which 3 were not cooled and were excluded. The gestational age and birth weights of cases and controls were comparable. The mean left ventricular global longitudinal strain (GLS) from apical 4 chamber view was noted to be significantly impaired in the asphyxiated infants (– 11.01% ± 2.48 vs – 21.45% ± 2.74, p <0.001). Cardiac output was significantly lower in the asphyxiated infants (97 ± 26 vs 230 ± 60 ml/kg/min). In asphyxiated infants, GLS correlated positively with cardiac output (r^2^ = 0.86, p< 0.001) and negatively with serum troponin levels (r^2^ = 0.64, p< 0.001). GLS was less impaired in infants on inotropes compared to those not on inotropic support, -12.55% (1.9) vs -10.2% (1.3), p= 0.018. Infants who died had a lower global strain value compared to survivors, – 9.7% (1.6) vs – 12.8% (2.6), p = 0.02.

**Conclusions:**

2D Speckle derived strain was impaired in asphyxiated infants. Significant correlations between GLS and cardiac output and troponin were noted.

## Background

Speckle tracking echocardiography (STE) is a relatively new technique for assessing neonatal cardiac function though it has been used in adult studies previously [1]. Strain, i.e. deformation, is expressed as either a fraction or the percentage change from the original dimension while strain rate (SR) is the deformation per unit time [1]. Quantitative assessment of myocardial function on the basis of STE utilising automated functional imaging (AFI) has advantages such as non-dependence on the angle of insonation and real time assessment in multiple dimensions [2]. There is emerging data in neonates on the applicability and usefulness of both DTI and STE derived strain analysis [3,4]. More recently, Singh et al demonstrated the use of STE derived right ventricular global longitudinal strain (GLS) in predicting clinical outcomes in preterm infants [5]. Normative myocardial deformation data on healthy neonates using 2D STE has not been described previously.

Approximately 1–2 newborns per 1000 live births could be affected by severe perinatal asphyxia [6] which can lead to multi-organ insult including cardiovascular dysfunction. Asphyxiated infants are managed with therapeutic hypothermia which has been shown to improve survival and neurological outcomes. Cardiovascular involvement in these infants can be demonstrated by changes in echocardiographic parameters and/or elevation in biochemical markers [6,7]. In a study on 7 asphyxiated infants treated with hypothermia, cardiac output was noted to be lower during the cooling phase [6]. Myocardial contraction has been traditionally described as fractional shortening and is used as a measure for global cardiac function. Some studies in the asphyxiated infants have shown a reduced fractional shortening [8] while others have not [3,7]. A new method for the evaluation of neonatal myocardium in the severely asphyxiated infants could be useful.

Serum cardiac troponin (cTnT) has also been studied as a marker of myocardial injury in neonates [7]. A value of >0.1 ng/ml (equivalent to 0.1 μg/L) has been proposed as a reliable measure of myocardial injury in neonates. The primary objective of this study was to determine myocardial strain indices using STE in term neonates with severe perinatal asphyxia and to compare them with healthy term controls. The secondary objective was to ascertain correlations between left ventricular GLS and left ventricular output (LVO) and cTnT (biochemical marker of myocardial injury) in the asphyxiated infants.

## Methods

Unit electronic database was accessed to identify infants with severe perinatal asphyxia administered therapeutic hypothermia during the period from January 2010 till August 2012. Demographic details and clinical data were retrieved and the archived echocardiographic images were analysed using AFI software (GE Echopac™). Echocardiographic monitoring of infants with clinical haemodynamic compromise is practised in the Unit since 2009 and has been approved as a quality improvement activity by the institutional ethics committee. One echocardiogram was done by echo-technologists from paediatric cardiology department; all others were done by investigator (AS). The information from the asphyxiated neonates was analysed retrospectively. Normative data collection from healthy term neonates was done prospectively and was approved as a separate study by the institutional ethics committee. At the institution, a daily list of deliveries is generated. The healthy controls in the postnatal ward were approached for participation in the study. The usual practice is for these infants to be discharged with mothers after 2 days (for vaginal deliveries) or 4–5 days for caesarean section deliveries. Data was analysed using software SPSS v18 and is presented as mean ± standard deviation and percentages for parametric and non-parametric data. Correlations between variables were assessed by Pearson’s coefficient of correlation while data between the cases and controls was compared using two tailed Unpaired Student-t test. Significance was set at p< 0.05. The intraobserver repeatability was analysed by calculating the intraclass correlation coefficients. This is an estimate of the reliability of the measurement and varies from 0 (no reliability) to 1 (total reliability, when test = retest measure). The intraobserver variability was assessed by performing off-line analysis on the same patients one week apart. The observer was blinded to the categorization of the infant.

### Subject population

Therapeutic hypothermia is the standard of care for asphyxiated infants in Australia and New Zealand. The infants are cooled when all 3 of the following criteria are met:

a) Gestational age > 35 weeks and birth weight > 2000 g

b) At least 2 of the following

• Apgar score ≤ 5 at 10 minutes and/or

• Mechanical ventilation or need for resuscitation at 10 minutes and/or

• Cord pH < 7.0 or arterial/venous blood gas pH < 7.0 or base deficit ≥ 12 on sample obtained within 1 hour of birth

c) Moderate or severe hypoxic ischaemic encephalopathy (Sarnat’s & Sarnat’s staging).

The aim is to maintain the core body temperature between 33–34°C for the first 72 hours and achieve rewarming over a period of 12 hours. None of the infants in this study received sedation or were paralysed.

### Echocardiographic measurements

**Figure 1:**
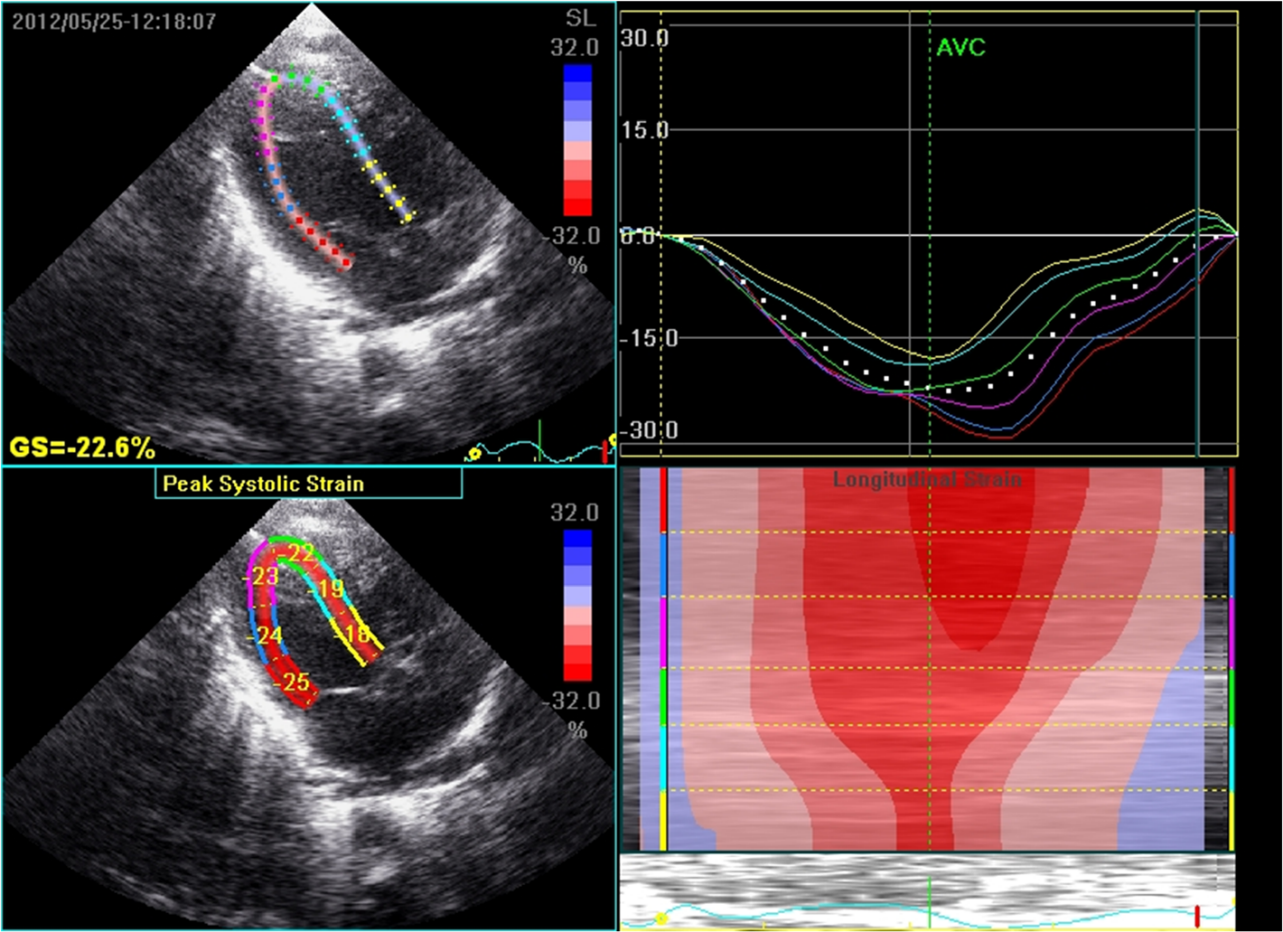
**Top left: mapping of the region of interest in the LV myocardium.** Bottom left: peak segmental longitudinal strain values. Top right: Segmental peak systolic strain curves. Bottom right: curved anatomical M-mode of longitudinal deformation.

All echocardiographic studies were done using GE Vivid 7 equipment (GE Vingmed Ultrasound, Horten, Norway) and 10 Hz probe. Real time 2D data on the first postnatal day was collected from an apical 4 chamber view in all the subjects and stored digitally for offline analysis on the *Echopac* system. Gain settings were optimized to obtain as clear differentiation as possible between the ventricular cavity and the wall. Endocardial tracing over a single frame was manually drawn for the LV. This was followed by automatic tracking of endocardial borders throughout the cardiac cycle. The LV was divided into six segments: basal septal, middle septal, apical septal, apical lateral, middle lateral and basal lateral (Figure 1). Segmental strains as well as GLS were measured as displacement of speckles in relation to one another along the endocardial contours throughout the different phases of the cardiac cycle. The negative SR values in the apical view depict longitudinal shortening in systole. The timing of aortic valve closure was defined from the pulse Doppler recording from the apical 4-chamber view and displayed automatically. Based on the aortic valve timing, Echopac algorithm generates 7 curves that represent strain/strain rate in the longitudinal direction (along the heart wall) for 6 specific myocardial segments and one global value representing combined strain from all segments. A frame rate of at least 80 was used for storage and analysis. We attempted image analysis for both longitudinal (apical 4 chamber) and radial (short axis) measurements. While this was possible for all apical 4 chamber views, inadequate image quality for short axis in 6 of 21 prevented further analysis. The intra-observer variability analysis was presented in the form of intra-class correlation for longitudinal (apical 4 chamber) measurements.

### 2D Doppler Ventricular performance

LVO was measured from the apical 5 chamber view. Ventricular output was calculated in ml/kg/min by the formula (Velocity Time Integral × [π × outflow diameter^2^/4] × heart rate)/body weight. The angle of insonation was kept to < 15°. The cross sectional area was uniformly measured at the valve hinges. Diastolic performance was evaluated from Doppler interrogation of transmitral flow. Specifically, the peak velocity of early passive wave (E wave) and late active wave (A wave) were measured to calculate the E/A ratio [9]. LV dimensions for fractional shortening were measured using M mode from parasternal long axis view at the level just distal to the mitral valve leaflet tips at end diastole.

## Results

Twenty four infants with severe perinatal asphyxia were admitted during the study period, of whom 7 died. Three infants were not cooled and were excluded from the analysis (one—33 weeks, two—severe coagulopathy and pulmonary hypertension requiring nitric oxide). The likely aetiology in 17/21 cases was meconium aspiration syndrome. Two infants were born in the setting of antepartum haemorrhage while one had a tight cord around the neck. Exact cause of asphyxia in one was not known. Table 1 depicts the demographic and clinical characteristics of the study population. The asphyxiated infants had a low initial pH and high serum lactate. The start of cooling ranged from 1–5 hours and the median (range) duration was 72 h (6–72 hours). Serum cTnT values were done in 18 infants on the same day as echocardiograms. These were high (1. 71 ± 0.66 μg/L) compared to the normal reference range for the local laboratory (<0.08 μg/L). Troponin levels were not measured in the control population. At the time of the echocardiogram, only one infant was hypotensive with a mean arterial pressure of 33 mmHg, while seven infants were already on inotropes.

**Table 1:** Demographic and baseline clinical characteristics of the study populations

**Variable**	**Asphyxiated infants**	**Healthy term controls**
	**(n=21)**	**(n=21)**
Gestational age (weeks)	39 ± 1.3	39 ± 1.4
Birth weight (g)	3807 ± 197	3812 ± 176
Inotropes dosage median (range)		
Dobutamine (n=10)^	10 (6, 10)	
Dopamine (n=3)*^	10 (10, 10)	
Timing of echocardiograms		
median (range) hours	6.2 (3-11)	32 (26, 40)
Blood gas parameters		Not applicable
pH	6.96 ± 0.13	
Base deficit	-18.1 ± 8.9	
Serum lactate (mmol/L)	20.3 ± 2.5	
Ventilation (at the time of echo)		Not applicable
Mode (conventional), n (%)	21 (100)	
MAP (cmH_2_O)	8.1 ± 0.9	
FiO_2_^	0.26 (0.21, 0.32)	

*in addition to Dobutamine, pH mostly from cord arterial gas, ^-median (range).

**Figure 2:**
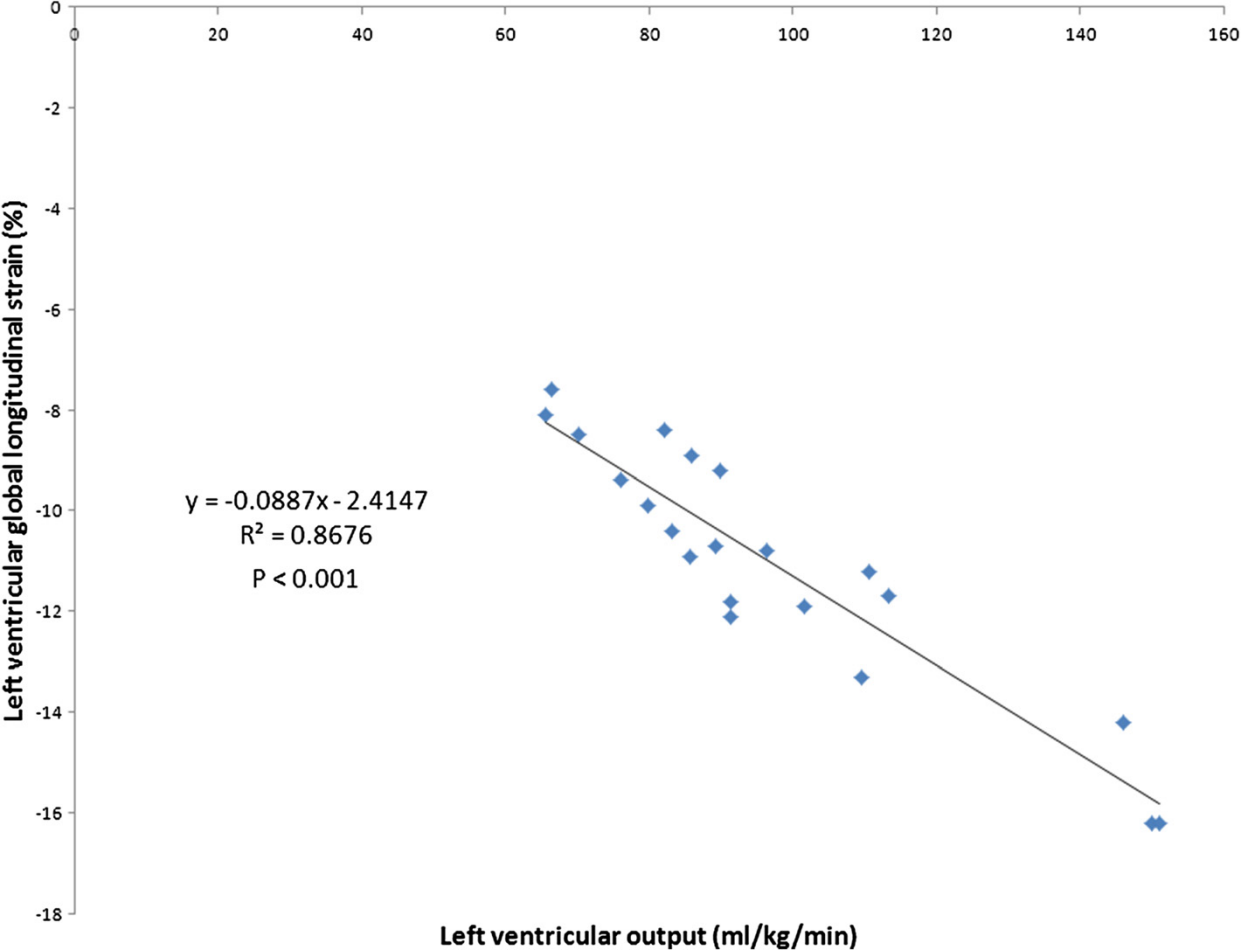
Correlation between global longitudinal strain and left ventricular output in asphyxiated infants.

**Figure 3:**
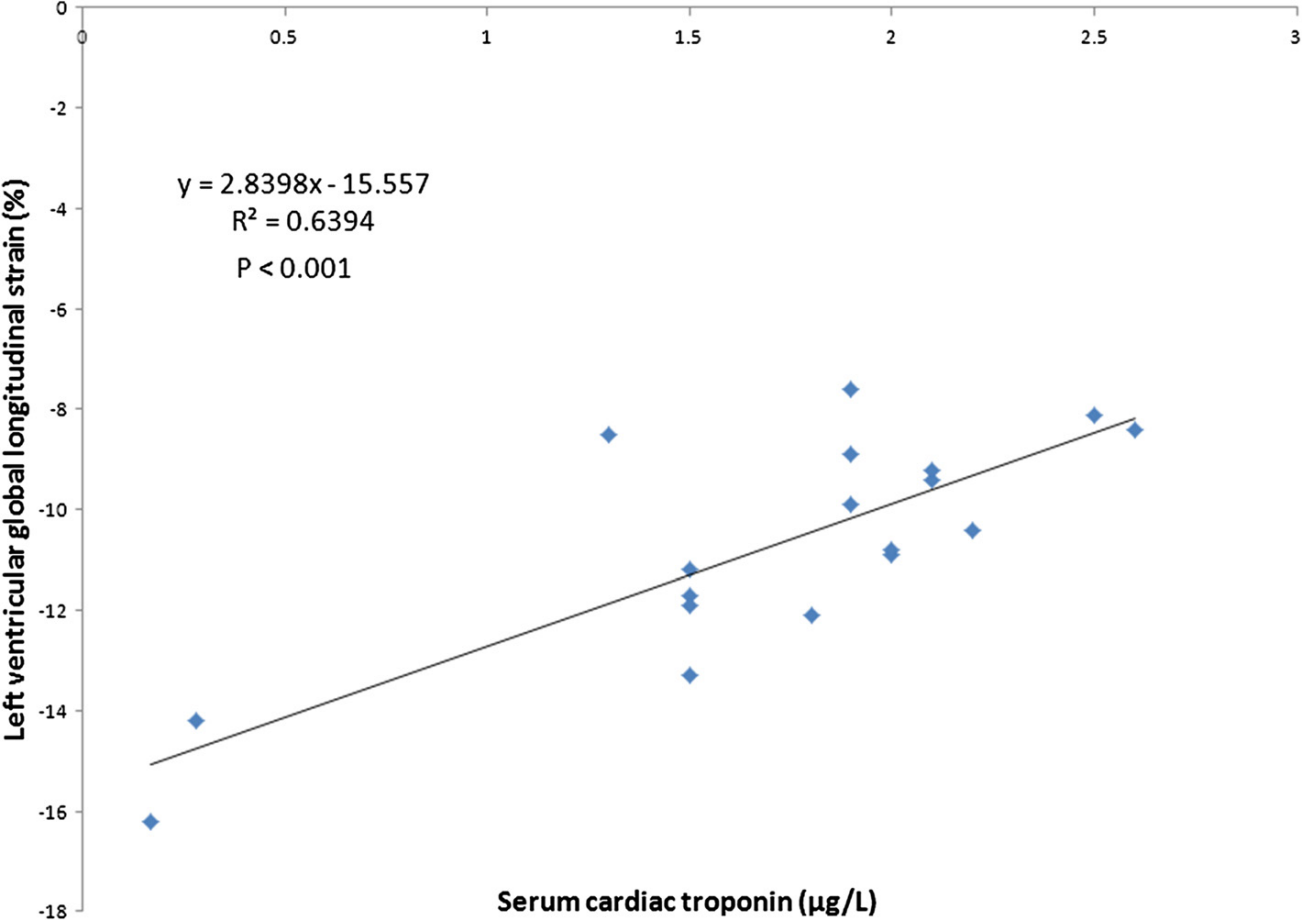
Correlation between global longitudinal strain and serum cardiac troponin.

**Figure 4:**
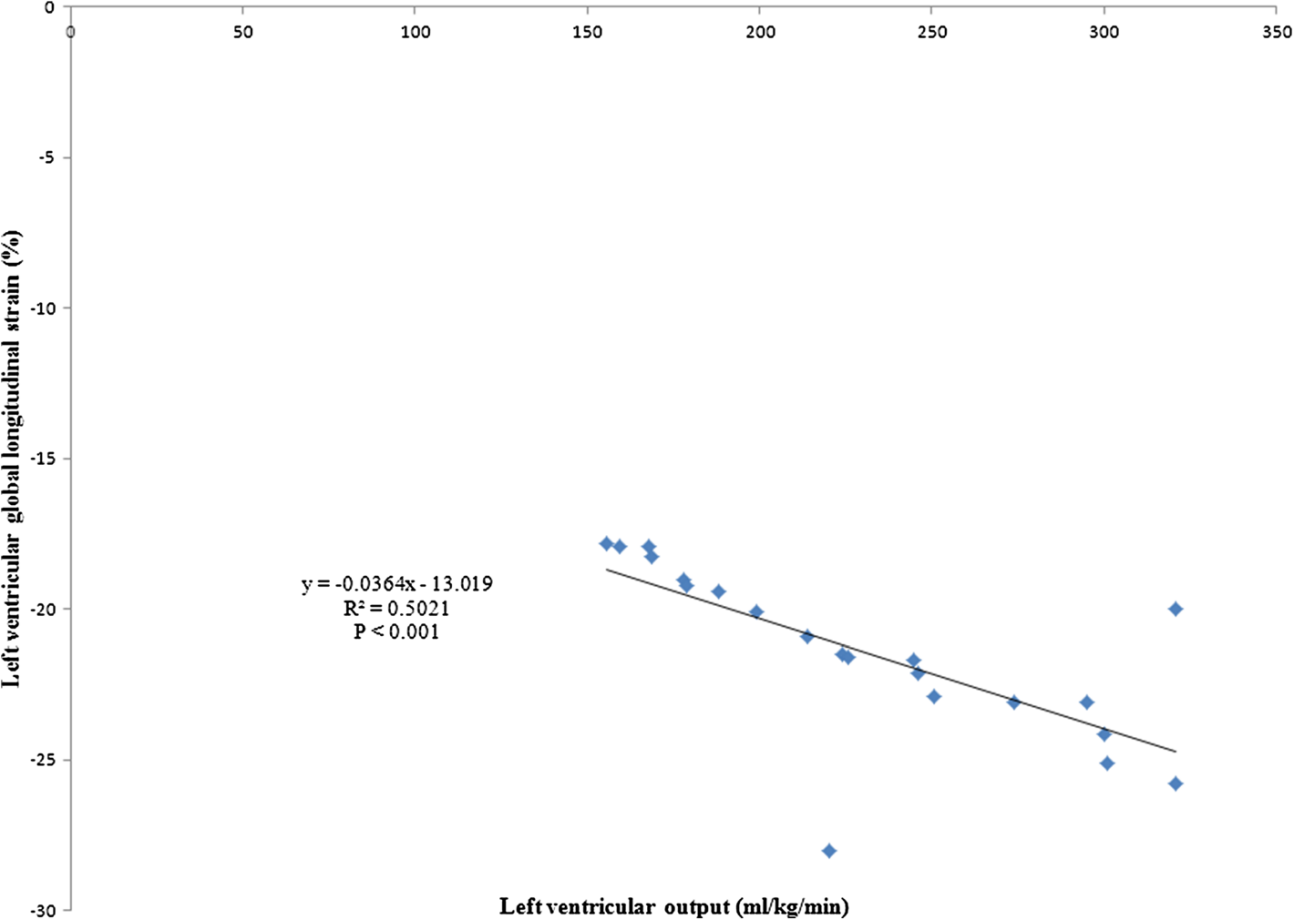
Correlation between global longitudinal strain and left ventricular output in control infants.

The mean LV GLS was noted to be significantly impaired in the asphyxiated infants (– 11.01% ± 2.48 vs – 21.45% ± 2.74, p <0.001). Table 2 depicts speckle derived LV peak systolic segmental strain and SR in the asphyxiated infants and healthy term controls in the basal, middle and apical segments in the apical view. Segmental strain and SR were both noted to be significantly lower in the asphyxiated infants; however, no significant segmental gradient was noted. Table 3 depicts conventional Doppler and 2D indices in the respective groups. The ductus arteriosus was noted to be patent in a proportion of infants. For control infants, it shunted left to right in all infants while for the asphyxiated infants, it shunted left to right in 11 and mixed in 2; always less than 30% of cardiac cycle. Cardiac output was lower in the asphyxiated infants and the LV GLS correlated positively with cardiac output and negatively with serum cTnT (Figures 2 and 3). GLS correlated with LVO in the control infants as well (r^2^ = 0.5, p < 0.001, Figure 4). Seven asphyxiated infants were on inotropes at the time of the echocardiogram. GLS in these infants was higher compared to those not on inotropic support, -12.55% (1.9) vs -10.2% (1.3), p= 0.018. Eight of 21 infants died (38%). Global strain in the infants who died was lower compared to survivors, – 9.7% (1.6) vs – 12.8% (2.6). The intraclass correlation coefficient for intra-observer echocardiographic measurements are presented in Table 4.

**Table 2:** Comparison of left ventricular peak longitudinal systolic strain and strain rates (SR) in the apical 4- chamber view

	**Basal septal strain (%)**	**Basal septal SR (/s)**	**Middle septal strain (%)**	**Middle septal SR (/s)**	**Apical septal strain (%)**	**Apical septal SR (/s)**	**Basal lateral strain (%)**	**Basal lateral SR (/s)**	**Middle lateral strain (%)**	**Middle lateral SR (/s)**	**Apical lateral strain (%)**	**Apical lateral SR (/s)**
Asphyxiated infants (n=21)	−13.6±2.48	−1±0.44	−12.5±1.25	−1.1±0.1	−13.3±1.6	−1.12±0.1	−12.8±1.46	−1.08±0.09	−13.2±1.75	−1.1±0.12	−13.61±1.6	−1.09±0.1
Controls (n=21)	−24.5±2.36	−1.88 ±0.25	−24.14±2.9	−1.86±0.24	−24.75±2.2	−1.8±0.2	−24.6±2.14	−1.71±0.17	−24.3±1.58	−1.71±0.24	−24.6±1.8	−1.62±0.18

P value for all <0.001.

**Table 3:** 2D and conventional Doppler indices in the asphyxiated and control infants

**Variable**	**Asphyxiated infants (n=21)**	**Healthy term controls (n=21)**	
Temperature (°C)	33.5 ± 0.5		
Fractional shortening (%)	26.4 ± 5.5	33.5 ± 5.3	<0.0001
Aortic stroke volume (ml/kg)	0.8 ± 0.3	1.6 ± 0.2	< 0.001
Left ventricular output (ml/kg/min)	97 ± 26	230 ± 60	< 0.001
Mitral E/A ratio	1.37 ± 0.2	0.85 ± 0.06	< 0.001
Transductal diameter (mm)	0.7 ± 0.2*	0.8 ± 0.2**	NS

*n=13, **n=14, *NS* not significant.

**Table 4:** Intra-class correlation and coefficient of variation for various echocardiographic parameters

**Echocardiographic parameter**	**Study group**	**Intra-class correlation coefficient**	**Coefficient of variation (%)**
Global longitudinal strain	Cases	0.85	7.5
	Controls	0.83	8.1
Strain rate	Cases	0.76	9.1
	Controls	0.79	8.8
Stroke volume	Cases	0.82	8.3
	Controls	0.8	8.4
Fractional shortening	Cases	0.8	8.6
	Controls	0.82	8.1
Transmitral Doppler	Cases	0.85	7.1
	Controls	0.82	7.8

## Discussion

Speckle tracking echocardiography is a relatively recent addition to neonatal non-invasive cardiac diagnostics and this study presents data on its application in asphyxiated infants. LV GLS was noted to be significantly impaired in asphyxiated infants. No significant segmental gradient was noted. Global strain was associated with a low cardiac output state and elevated cTnT.

### Acute myocardial ischemia and strain imaging

Myocardial deformation is affected by complex interactions between inherent cardiac contractile force and extrinsic loading (both pre and after) conditions. Hence, both could determine the pattern and the magnitude of deformation. Impairment in deformation could affect both shortening and lengthening. In acute ischemic conditions, the affected myocardial tissue does not demonstrate shortening or lengthening activity and it reflects in reduced systolic SR or strain. The deformation data related to ischemia and infarction is predominantly from adult literature and used DTI strain analysis. Our study used STE to assess asphyxiated infants. These infants were already severely haemodynamically compromised which reflected in abnormal conventional parameters of fractional shortening, stroke volume and LVO. While the numbers in each group were small, infants on inotropes had greater global deformation compared to those who were not on inotropes. There is limited data on the effect of inotropes on myocardial deformation which has been studied using DTI. In porcine myocardium, the peak systolic strain rate increased with incremental dobutamine challenge [10,11]. Using the 2D speckle tracking analysis on healthy adults, a dose-response relationship to dobutamine was demonstrated previously [12]. Strain and SR gradually increased with low doses of dobutamine. In our study, lower GLS was also associated with higher mortality. While the cause of death could not be ascribed solely to cardiovascular issues, it could be, in our opinion, a marker of severity of the overall disease process.

Czernik and colleagues used STE to assess evolution in cardiac performance during various phases of therapeutic hypothermia in 8 asphyxiated neonates [4]. Assessments were made at 4 time points; start (T1) and end of hypothermia (T2), immediately after rewarming (T3) and the age of 5 to 7 days (T4). The GLS was impaired in the T1 analysis which showed a significant increase at the end of the first week. While this study did not include a control group, it did provide additional information about cardiac adaptation to disease process and hypothermia. The timing of the first echocardiographic evaluation in this study was different from ours.

### Correlation between various cardiac indices

Myocardial deformation is an angle independent measurement if the speckle tracking technique is based on 2D gray ultrasound image acquisitions. Conventional Doppler derived indices (such as LVO), on the other hand, typically depend on the angle of insonation. Given that a third of the patients were on inotropes, it might under estimate the severity of the haemodynamic compromise. We noted a significant correlation between LV GLS and LVO in both groups and between GLS and cTNT in the asphyxiated infants. The latter remains the best marker of acute heart injury with high predictive values [13]. The DTI derived longitudinal systolic SR has previously been shown to correlate with global measures of systolic function in anesthetized mongrel dogs and humans [14,15]. Changes in deformation closely correlated to changes in contractility (best with changes in stroke volume). Belghitia et al evaluated the accuracy and reproducibility of GLS in determining LV function in comparison to reference echocardiographic and angiographic methods [16]. Strong correlations were observed with a high level of reproducibility demonstrating its clinical applicability, short acquisition time, feasibility and accuracy in patients admitted with myocardial infarction.

2D strain analysis has been shown to be useful in neonatal settings as well. El-Khuffash et al recently demonstrated feasibility and reliability of STE in premature infants with a haemodynamically significant ductus arteriosus [17]. Echocardiography was performed in 19 preterm infants before, 1 hour after and 18 hours after surgical duct ligation. GLS values significantly decreased 1 hour after but had significantly improved 18 hours after the procedure. Singh and colleagues assessed RV myocardial strain and SR in premature infants with chronic lung disease, to determine if measurements done at 32 weeks gestation, predict need for respiratory support at 36 weeks corrected gestational age [5]. Right ventricular GLS and SR were noted to be significantly lower at 32 weeks for the infants who still required respiratory support at 36 weeks and continued to be lower at 36 weeks corrected age. The authors postulated that early RV dysfunction contributes to, and identifies premature infants who are at risk for, persistent cardiorespiratory dysfunction.

As with all new echocardiographic methods, feasibility and variability are key areas for investigators. Feasibility of STE has been previously demonstrated in neonates and adults [4,16,17]. Image optimization during acquisition and use of gain settings allow better mapping of the region of interest and are important for low variability.

## Limitations

While the small number of subjects as a limitation is accepted, this study is preliminary work describing STE in critically sick neonates. We only depicted the apical 4- chamber approach which enables assessment of the interaction of inferolateral and anteroseptal segments simultaneously; radial and circumferential measurements could give additional information. Since therapeutic hypothermia is standard of care in the region, we did not have a population of asphyxiated infants who were not cooled for comparison with the study population. Lack of serial measurements as a limitation is accepted.

## Conclusions

Myocardium of asphyxiated infants showed impaired global and segmental deformation as well as rate of deformation during systole compared to healthy term neonates. Deformation indices correlated with 2D Doppler derived indices of cardiac output in both groups. While the conventional parameters remain the cornerstone of echocardiographic assessments, speckle tracking is an upcoming research tool which has shown promise in various neonatal clinical situations. We believe these measurements could potentially be useful in the management of haemodynamically unstable infants, especially those needing inotropic support; however, additional research is required.

## Abbreviations

SR: Strain rate; AFI: Automated functional imaging; DTI: Doppler tissue Imaging; cTnT: cardiac troponin; LVO: Left ventricular output; GLS: Global longitudinal strain.

## Competing interests

The authors declare that they have no competing interests.

## Authors’ contributions

AS- Contribution to study design and conception, data acquisition and analysis, drafting the manuscript. Is the corresponding author. FW-Critical revision and drafting manuscript, with data interpretation. SM-Design and conception with critical review on the topic. All authors read and approved the final manuscript.

## References

[B1] Pislaru C Abraham TP Belohlavek M Strain and strain rate echocardiography Curr Opin Cardiol 2002 17 443 454 10.1097/00001573-200209000-00002 12357119

[B2] Pirat B McCulloch ML Zoghbi WA Evaluation of global and regional right ventricular systolic function in patients with pulmonary hypertension using a novel speckle tracking method Am J Cardiol 2006 98 699 704 10.1016/j.amjcard.2006.03.056 16923465

[B3] Nestaas E Støylen A Brunvand L Fugelseth D Longitudinal strain and strain rate by tissue Doppler are more sensitive indices than fractional shortening for assessing the reduced myocardial function in asphyxiated neonates Cardiol Young 2011 21 1 7 20923594 10.1017/S1047951109991314

[B4] Czernik C Rhode S Helfer S Schmalisch G Beuhrer C Left ventricular longitudinal strain and strain rate measured by 2D speckle tracking echocardiography in neonates during whole body hypothermia Ultrasound Med Biol 2013 8 39 1343 1349 23743104 10.1016/j.ultrasmedbio.2013.03.024

[B5] Singh GK Levy PT Holland MR Hamvas A Novel methods for assessment of right heart structure and function in pulmonary hypertension Clin Perinatol 2012 39 685 701 10.1016/j.clp.2012.06.002 22954276

[B6] Gebauer CM Knuepfer M Robel-Tillig E Pulzer F Vogtmann C Haemodynamics among neonates with hypoxic-ischemic encephalopathy during whole-body hypothermia and passive rewarming Pediatrics 2006 117 3 843 850 10.1542/peds.2004-1587 16510666

[B7] Szymankiewicz M Matuszczak-Wleklak M Hodgman JE Gadzinowski J Usefulness of cardiac troponin T and echocardiography in the diagnosis of hypoxic myocardial injury of full-term neonates Biol Neonate 2005 88 19 23 10.1159/000084067 15731551

[B8] Costa S Zecca E De Rosa G De Luca D Barbato G Pardeo M Romagnoli C Is serum troponin T a useful marker of myocardial damage in newborn damage in newborn infants with perinatal asphyxia? Acta Paediatr 2007 96 181 184 10.1111/j.1651-2227.2007.00104.x 17429901

[B9] Schmitz L Stiller B Pees C Koch H Xanthopoulos A Lange P Doppler-derived parameters of diastolic left ventricular function in preterm infants with a birth weight <1500 g: reference values and differences to term infants Early Human Development 2000 76 101 114 14757262 10.1016/j.earlhumdev.2003.11.003

[B10] Jamal F Strotmann J Weidmann F Kukulski T D’hooge J Bijnens B Noninvasive quantification of the contractile reserve of stunned myocardium by ultrasonic strain rate and strain Circulation 2001 104 1059 1065 10.1161/hc3501.093818 11524402

[B11] Gorcsan J III Deswal A Mankad S Mandarino WA Mahler CM Yamazaki N Quantification of the myocardial response to low dose dobutamine using tissue Doppler echocardiographic measures of velocity and velocity gradient Am J Cardiol 1998 81 1066 9514460 10.1016/s0002-9149(97)00973-9

[B12] Elmayergi NH Goodman JM Lee LS Sasson Z Are measures of left ventricular systolic performance during low dose dobutamine stress echocardiograms repeatable over time? Int J Cardiovasc Imaging 2013 Epub ahead of print 10.1007/s10554-013-0219-5 23589004

[B13] James S Armstrong P Califf R Simoons ML Venge P Wallentin L Troponin T levels and risk of 3 day outcomes in patients with the acute coronary syndrome: prospective verification in the GUSTO-IV trial Am J Cardiol 2003 115 241 244 10.1016/s0002-9343(03)00348-6 12935823

[B14] Yamada H Oki T Tabata T Iuchi A Ito S Assessment of left ventricular systolic wall motion velocity with pulsed tissue Doppler imaging: comparison with peak dP/dt of the left ventricular pressure curve J Am Soc Echocardiogr 1998 11 442 9 10.1016/S0894-7317(98)70024-0 9619616

[B15] Greenberg NL Firstenberg MS Castro PL Main M Travaglini A Odabashian JA Doppler-derived myocardial systolic strain rate is a strong index of left ventricular contractility Circulation 2002 105 1 99 105 10.1161/hc0102.101396 11772883

[B16] Belghitia A Brette S Lafitte S Reant P Picard F Serri K Automated function imaging: a new operator-independent strain method for assessing left ventricular function Arch Cardiovasc Dis 2008 3 163 169 18477943 10.1016/s1875-2136(08)71798-4

[B17] El-Khuffash A Jain A Dragulescu A McNamara PJ Mertens L Acute changes in myocardial systolic function in preterm infants undergoing patent ductus arteriosus ligation: a tissue Doppler and myocardial deformation study J Am Soc Echocardiogr 2012 25 1058 67 10.1016/j.echo.2012.07.016 22889993

